# Supplementary material for: A suppression-modification gene drive for malaria control targeting the ultra-conserved RNA gene mir-184
Source: Nat Commun. 2025 Apr 25;16:3923. doi: 10.1038/s41467-025-58954-5 (PMC12032250; doi:10.1038/s41467-025-58954-5)
Supplement: Supplementary file 1 — Supplementary Information [file 41467_2025_58954_MOESM1_ESM.pdf]

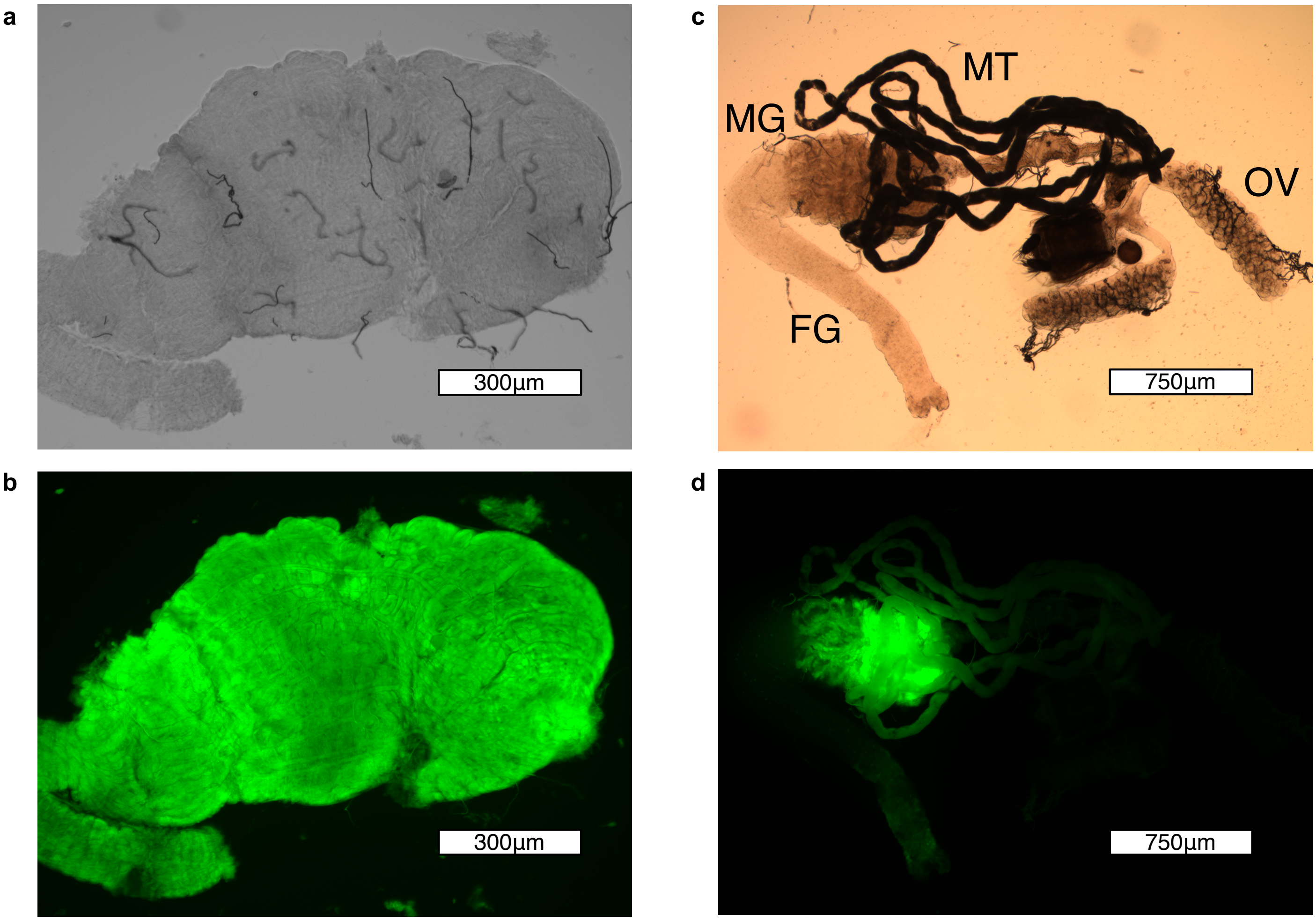

Supplementary Figure 1. Expression of eGFP in the midgut of miR-184^{D} mosquitoes. Transmission (**a**) and fluorescent (**b**) images of dissected adult female midgut 8 days post blood meal. The digestive and reproductive systems of dissected miR-184^{D} adult females were examined by transmission (**c**) and fluorescent (**d**) microscopy. FG: foregut; MG: midgut; MT: Malpighian tubules; OV: ovaries.

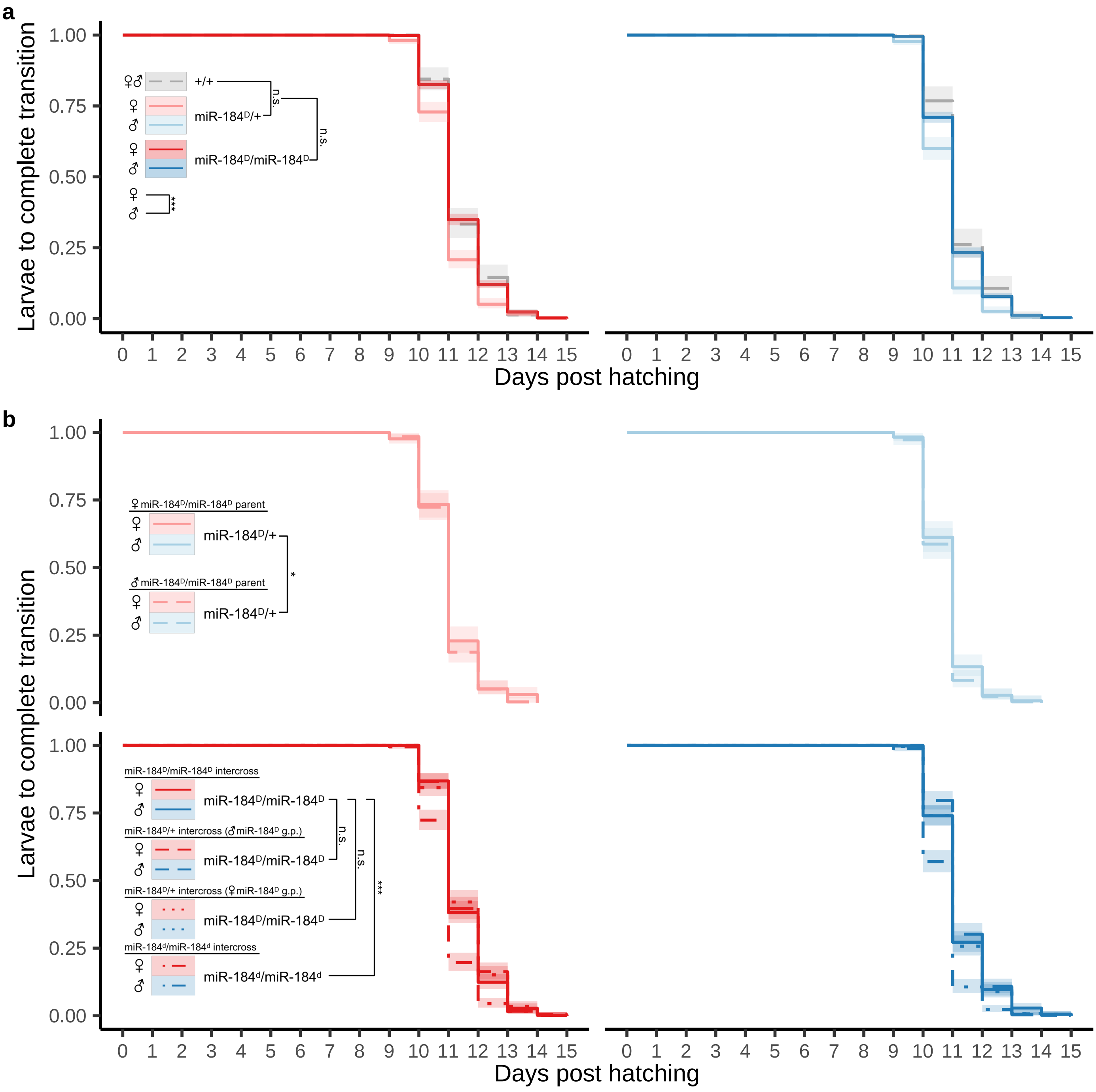

Supplementary Figure 2. Pupation rate of miR-184^{D} mosquitoes. a Averaged pupation time of female (left) and male (right) wild-type, miR-184^{D} hemizygous and miR-184^{D} homozygous larvae. Survival analysis was conducted using a mixed-effects Cox proportional hazards model. Pairwise comparisons averaged over the effect of sex were conducted using Tukey's method with P value adjustment. **b** Separate cross-conditions of the miR-184^{D} hemizygous (top) and miR-184^{D} homozygous groups (bottom). Individual contrasts were performed with multivariate t-distribution adjustment of *P* values (*P*^{ns}≥0.05, *P**<0.05, and *P****<0.001).

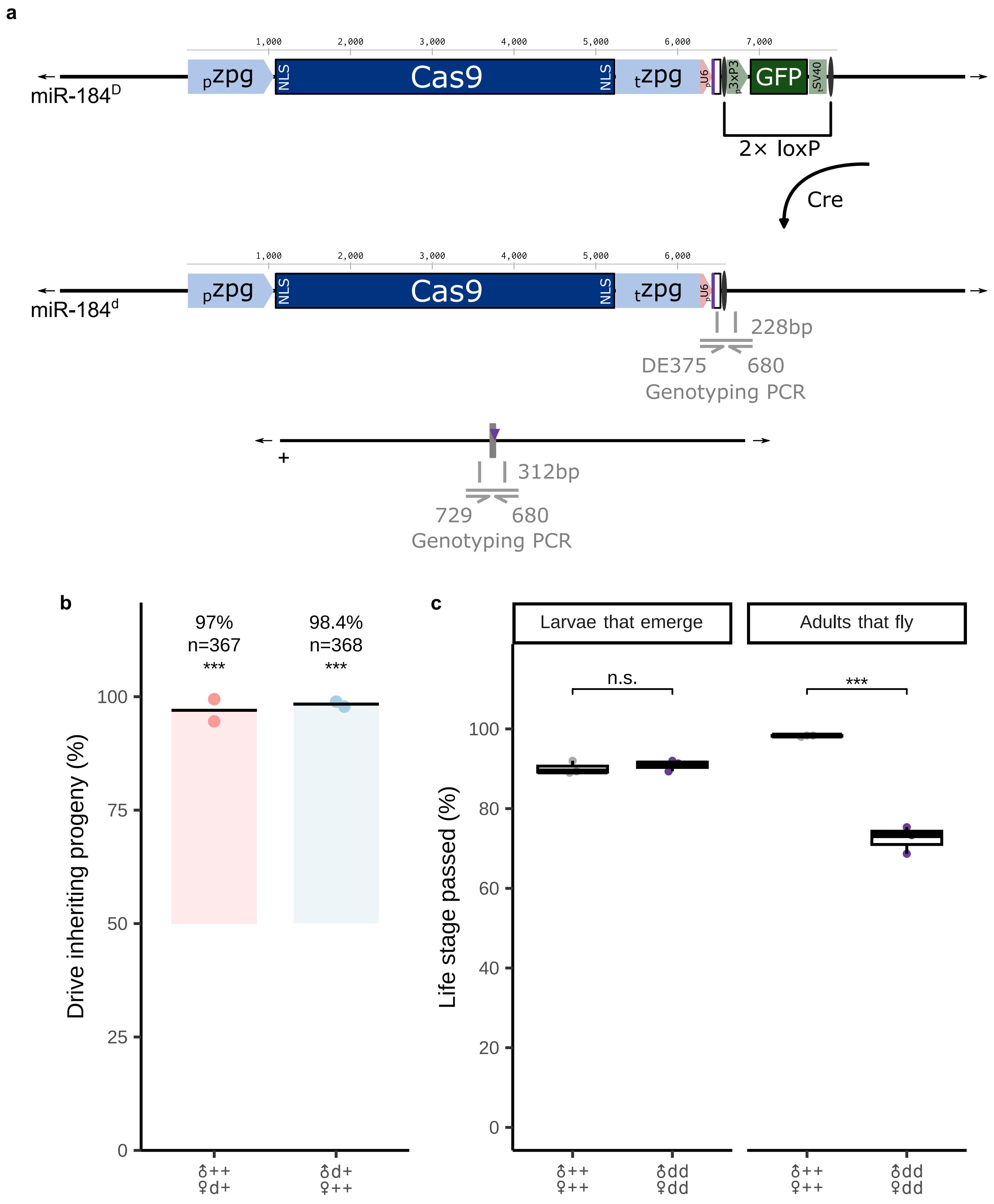

Supplementary Figure 3. The miR-184^{d} markerless drive strain. a Schematic showing the expected excision of the loxP flanked 3xP3-GFP-SV40 fluorescent reporter module following a cross with a Cre expressing strain. **b** Drive transmission in crosses of hemizygous miR-184^{d} transgenic males or females to the wild type. Each point shows the mean from a pooled independent biological replicate, with the inheritance rates over all replicates and the number of scored progeny indicated. Deviation from Mendelian inheritance rate was calculated using a generalised linear mixed model with replicate as a random effect (P^{***}<0.001). Bars originate at the expected inheritance rate and end at the observed mean inheritance. Inheritance rates of the GFP-negative miR-184^{d} element were scored by multiplexing PCR shown in a. **c** Developmental transition and adult flight ability of wild type and homozygous miR-184^{d} mosquitoes. Significance levels were calculated using a binomial GLM with replicates as a random effect (P^{ns}≥0.05 and P^{***}<0.001).

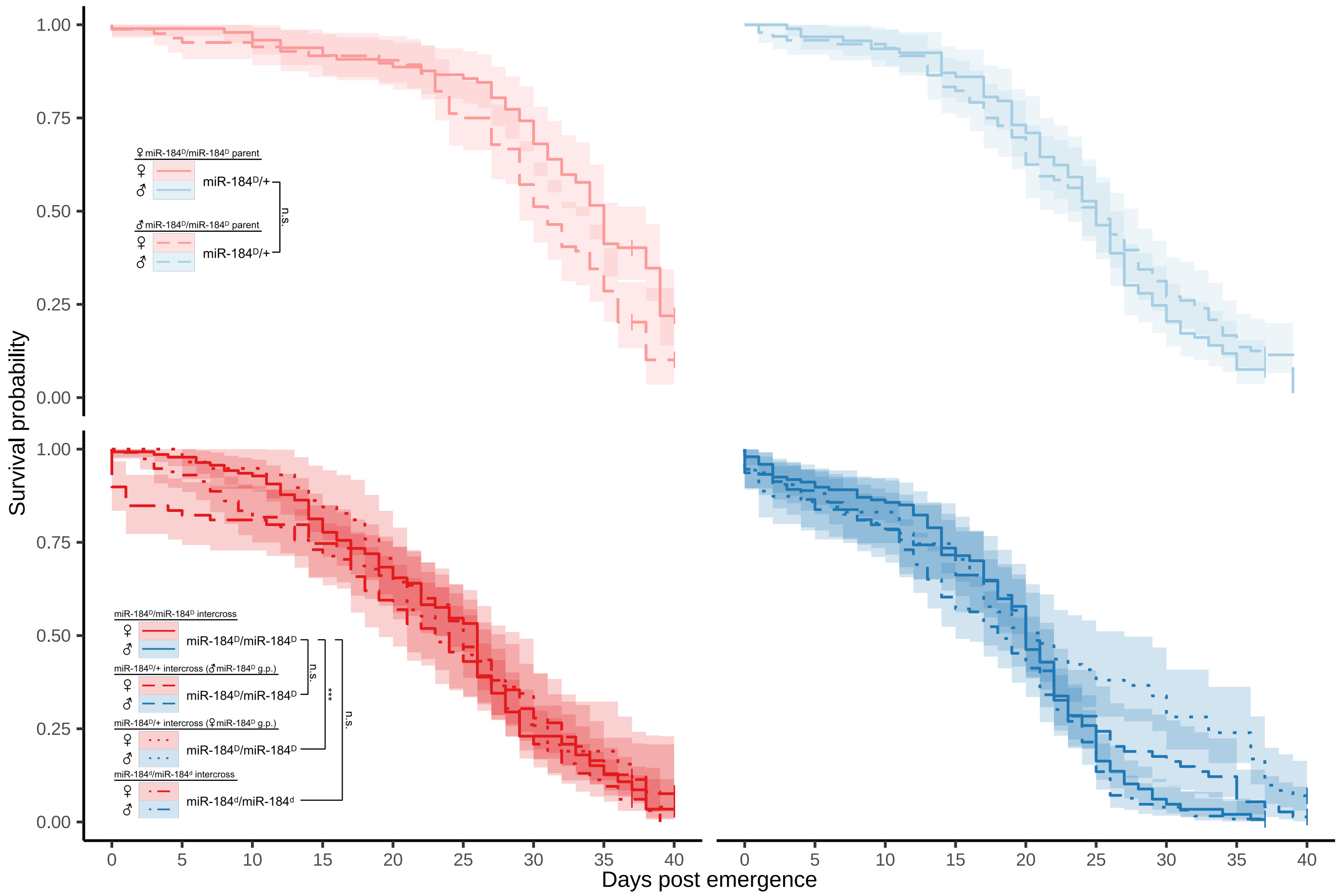

Supplementary Figure 4. Survival rate of adult miR-184^{D} mosquitoes. Separate cross-conditions of the miR-184^{D} hemizygous (top) and miR-184^{D} homozygous groups (bottom). Survival analysis was conducted using a mixed-effects Cox proportional hazards model. Individual contrasts were performed with multivariate t-distribution adjustment of *P* values (P^{ns}≥0.05 and *P****<0.001).

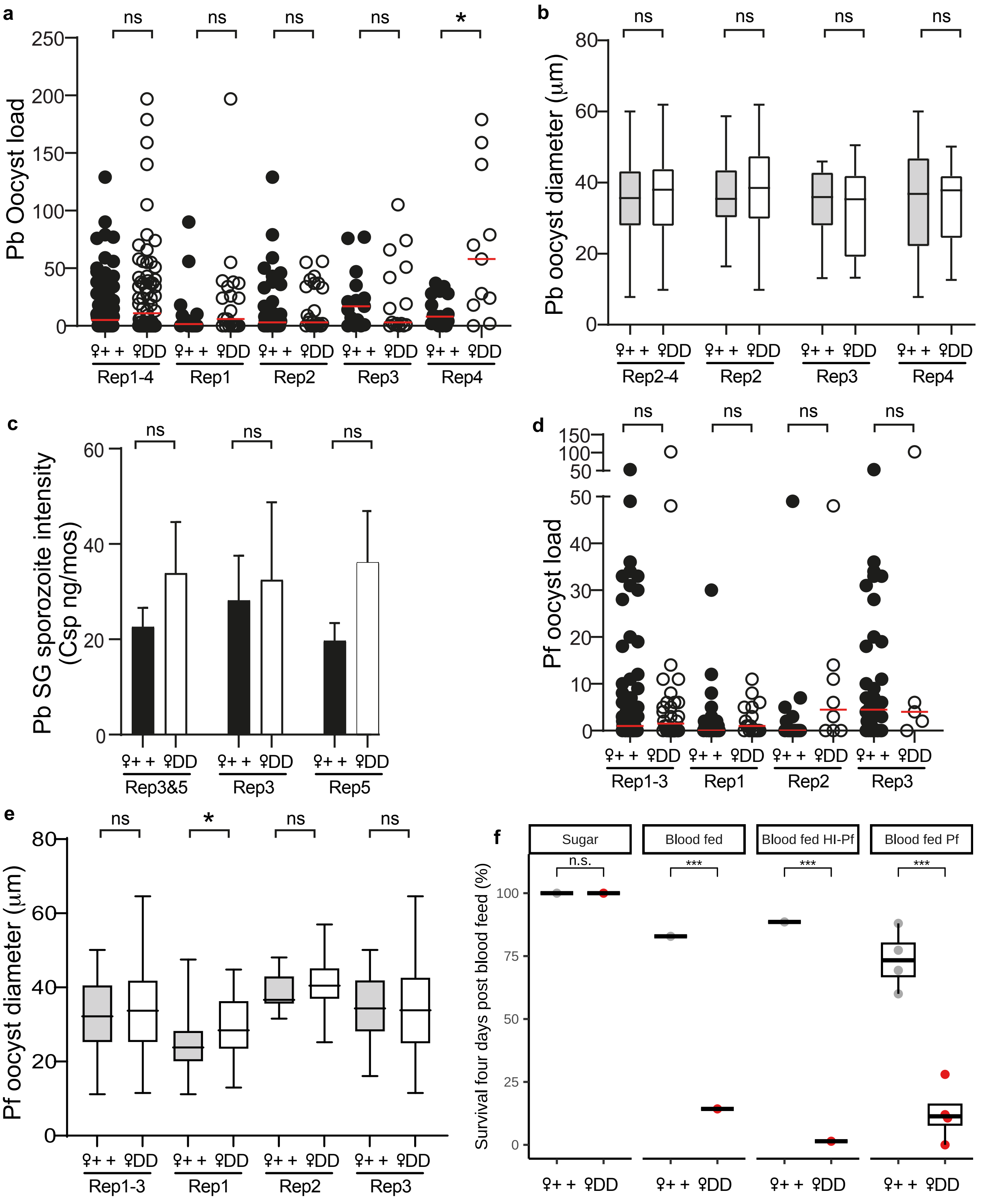

Supplementary Figure 5. *P. berghei* **and** *P. falciparum* **infection experiments. a** *P. berghei* oocysts load in the midguts of wild type and miR-184^{D} mosquitoes enumerated at 8 dpbf. (R1) or 12 dpbf. (R2-4) Individual biological replicates and the pooled data are presented. Red lines indicate the median. *P* values were calculated using the Mann-Whitney test ($P^{ns}\geq0.05$ and $P^{*}<0.05$). **b** Box plot of diameter measurements of oocysts in wild type and miR-184^{D} mosquitoes at 12 dpbf. Upper and lower whiskers represent the largest and smallest oocyst diameter, respectively. Horizontal line in each box indicates mean and whiskers show SEM. *P* values were calculated using the unpaired Student t-test ($P^{ns}\geq0.05$). **c** Average salivary gland sporozoite intensity with SEM at 19 dpbf measured by Circumsporozoite protein (CSP) quantitative PCR amplification. *P* values were calculated using the unpaired Student t-test ($P^{ns}\geq0.05$). **d** *P. falciparum* oocyst load in the midguts enumerated at 8 dpbf. (R1), 13 dpbf. (R2) and, 9 dpbf. (R3). *P* values were calculated using the Mann-Whitney test ($P^{ns}\geq0.05$). **e** Box plot of diameter measurements of *P. falciparum* oocysts at 8 dpbf. (R1), 13 dpbf. (R2) and, 9 dpbf. (R3). *P* values were calculated using the unpaired Student t-test ($P^{ns}\geq0.05$ and $P^{*}<0.05$). **f** Survival after 4 days of female wild type and homozygous miR-184^{D} mosquitos provided sugar, or an uninfected, heat-inactivated (HI), or *P. falciparum* infected blood-meal with a 2 day sugar withdrawal. Significance levels were calculated using a binomial GLM with replicates as a random effect ($P^{ns}\geq0.05$ and $P^{***}<0.001$).

a

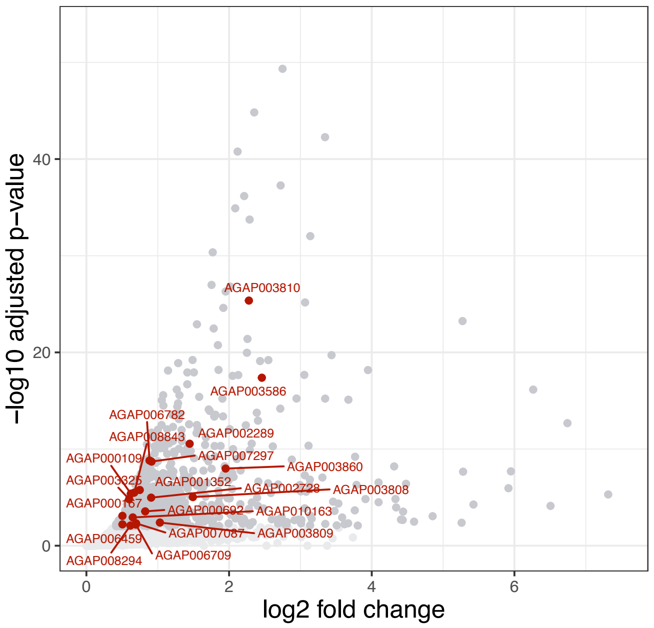

b

Domain	Term name	Term ID	Adjusted p-value
GO:CC	septate junction	GO:0005918	2.41603E-05
GO:BP	open tracheal system development	GO:0007424	2.8862E-05
GO:BP	respiratory system development	GO:0060541	2.8862E-05
GO:BP	regulation of tube size	GO:0035150	2.8862E-05
GO:BP	regulation of tube size, open tracheal system	GO:0035151	2.8862E-05
GO:BP	regulation of tube architecture, open tracheal system	GO:0035152	2.8862E-05
GO:CC	tight junction	GO:0070160	0.00020979
GO:CC	apical junction complex	GO:0043296	0.000713798
GO:CC	mitochondrion	GO:0005739	0.000960043
GO:BP	septate junction assembly	GO:0019991	0.00099991
GO:BP	tight junction assembly	GO:0120192	0.002387572
GO:BP	tight junction organization	GO:0120193	0.002387572
GO:BP	apical junction assembly	GO:0043297	0.002387572

c

```
Target: AGAP002289

length: 21

mfe: -22.7 kcal/mol
p-value: 1.000000e+00

Position: 2222

target 5'    G     UAAAUA        A 3'
              UAUCG      UCCGUCCA
              AUAGU      AGGCAGGU
miRNA  3' GGGA     CAAG            5'
```

```
Target: AGAP003808

length: 21

mfe: -30.7 kcal/mol
p-value: 1.000000e+00

Position: 671

target 5' G             GACU        G 3'
           CUCUUGUCGGUUC    UUCGUCUA
           GGGAAUAGUCAAG    AGGCAGGU
miRNA  3'
```

```
Target: AGA003809

length: 21

mfe: -25.2 kcal/mol
p-value: 1.000000e+00

Position: 8512

target 5' A   AA  GC   ACC         U 3'
           CCU  UG  UCA   CUCCGUCCA
           GGA  AU  AGU   GAGGCAGGU
miRNA  3' G            CAA           5'
```

```
Target: AGAP003810

length: 21

mfe: -23.4 kcal/mol
p-value: 1.000000e+00

Position: 1234

target 5'     G      AAUC  GUGAAGAAAAAGAA        A 3'
               AUCAGU    UC              UCCGUCCA
               UAGUCA    AG              AGGCAGGU
miRNA  3' GGGAA
```

Supplementary Figure 6. Analysis of putative miR-184 target genes in miR-184^{D} mosquitoes. a Differential upregulation of predicted miR-184 target genes based on the combined CLIP-seq datasets of Fu at al. 2020 and Dong et al. 2020 in the miR-184^{D} gut RNAseq dataset. The selection of genes with at least 5 unique miRNA:mRNA chimaeras and a minimum peak height of 5 reads under any condition yielded 133 high-confidence genes (69 and 49 genes from each datasets respectively), 20 of which were amongst the upregulated genes between miR-184^{D} and wild type mosquitoes ($P \leq 0.01$). These upregulated genes also present in the high-confidence CLIP-seq gene set are indicated in red. **b** Summary of enriched GO terms associated with significantly upregulated genes in the gut samples predicted to be miR-184 targets. **c** The miRNA-mRNA interactions predicted by RNAhybrid for 4 putative mir-184 target mRNAs in the GO:0005918 group. The miR-184 seed region is shown in red.

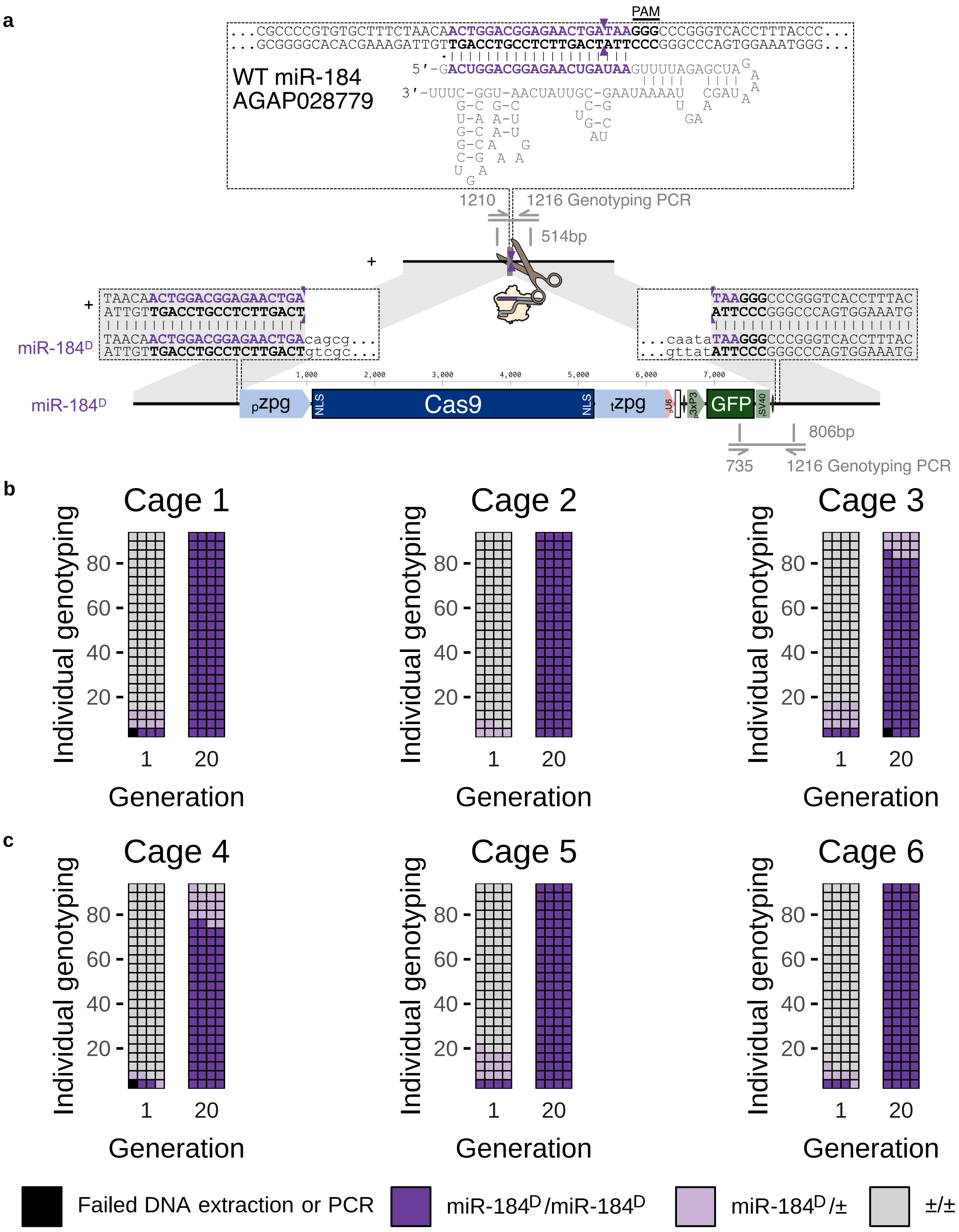

Supplementary Figure 7. miR-184^{D} gRNA target sequence context and genotyping PCR outcomes. a Illustration of the miR-184^{D} gRNA cut location and alignment of the cleaved wild-type DNA ends to the flanking sequence of the miR-184^{D} transgene. The positions of the primers used in genotyping generation 21 are indicated. Similar primers (729, 735, and 680) were used for genotyping generation 1 and 20 (not indicated). Each generation, the genotype of 92 individual larvae was assessed through multiplex PCR and size discrimination for the miR-184^{D} (**b**), and miR-184^{D} + MM-CP (**c**) cages. ± Indicates a PCR product from a non-drive allele which may be a wild type or resistance allele.

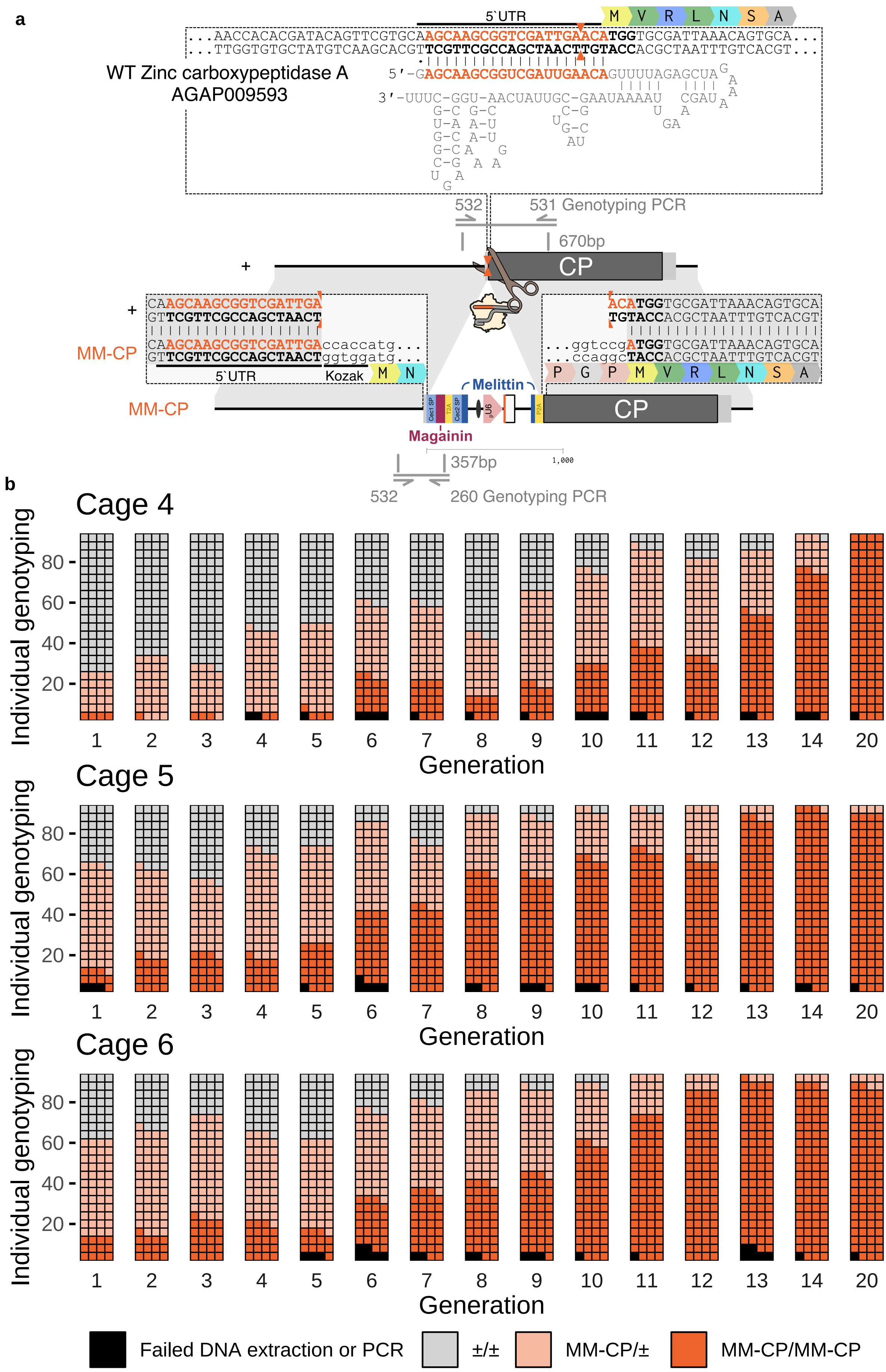

Supplementary Figure 8. MM-CP gRNA target sequence context and genotyping PCR outcomes. a Illustration of the MM-CP gRNA cut location and alignment of cleaved wild-type DNA ends to the flanking sequence of the MM-CP transgene. The positions of the primers used in genotyping generation 1-14 and 20-21 are indicated. Each generation, the genotype of 92 individual larvae were assessed through multiplex PCR and size discrimination for the miR-184^{D} + MM-CP (**b**) cages. ± Indicates a PCR product from a non-drive allele which may be a wild type or resistance allele.

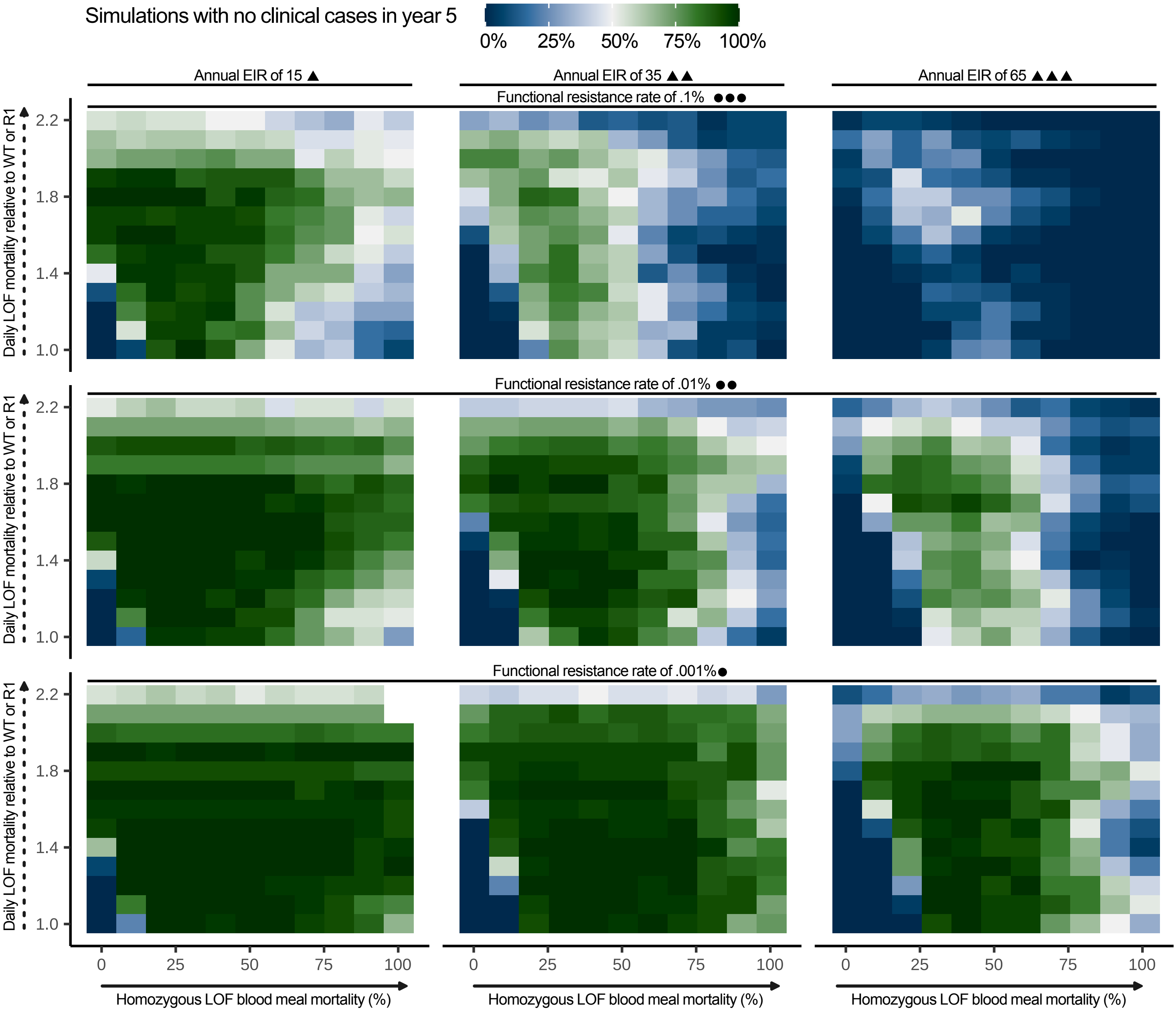

Supplementary Figure 9. Modelling gene propagation and its effect on the local malaria burden of 0-5 year olds. Heatmaps are coloured based on the probability of the elimination of the malaria burden in 0 to 5 year olds by the end of the 5th year following gene drive releases averaged across 40 stochastic simulations and did not allow for the importation of malaria from outside the release zone.

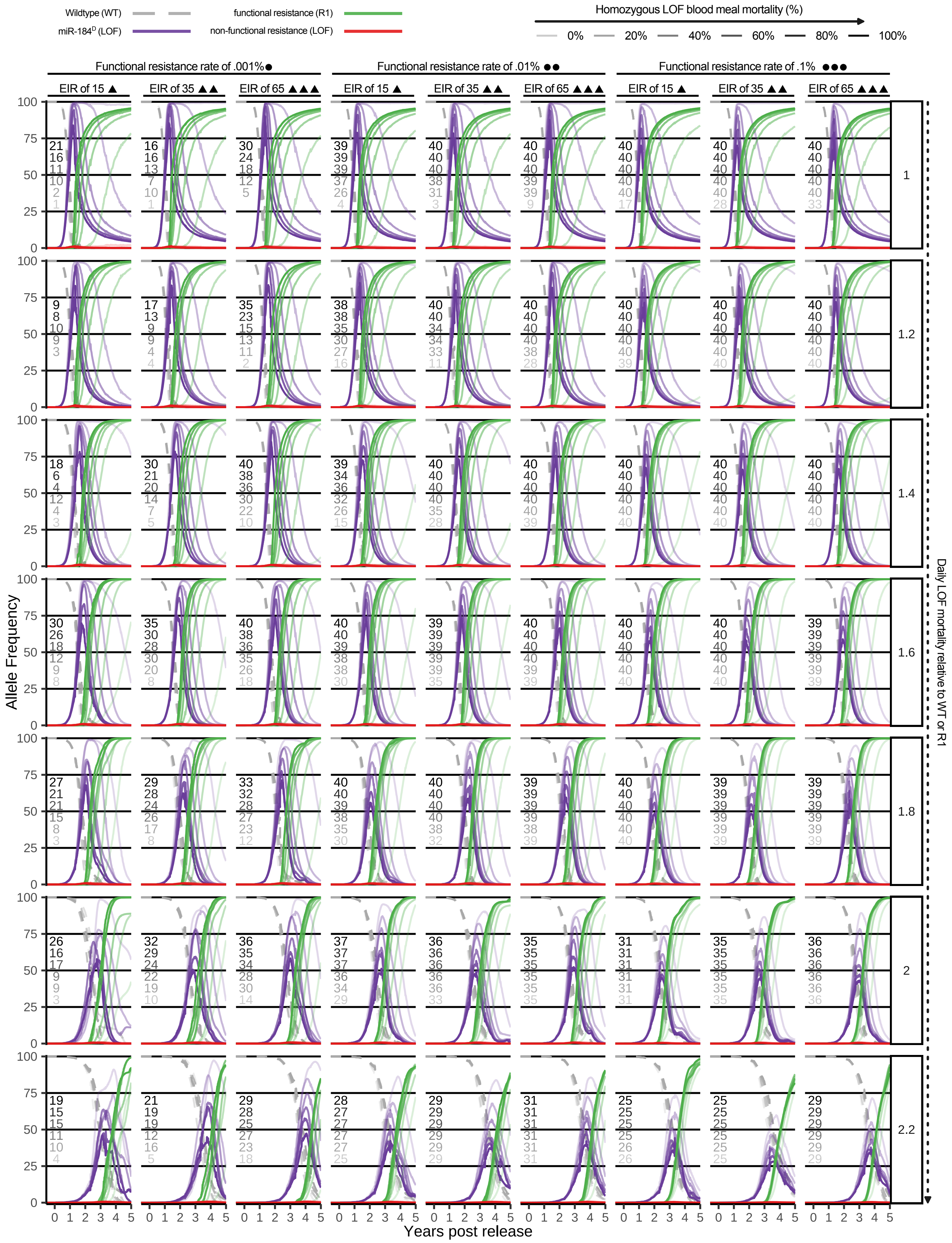

Supplementary Figure 10. Average allele frequency dynamics of simulations where functional resistance alleles predominated or persisted by the end of year 5. Each panel lists the number of simulations (out of 40) where functional resistance alleles were present 5 years after the release and for which the mean allele frequency is plotted. The opacity of the lines and text is indicative of the blood meal mortality rate for a given group of simulations.

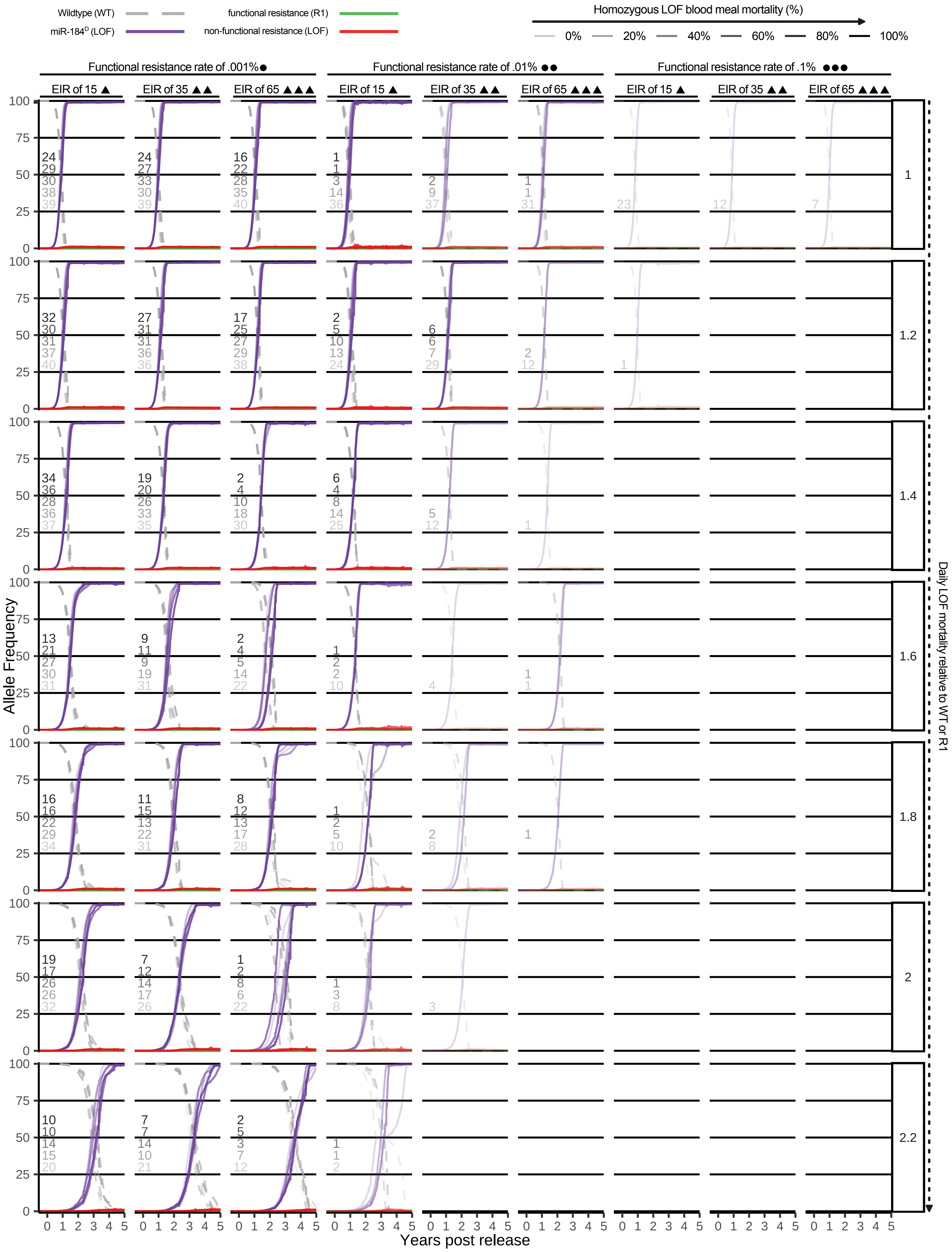

Supplementary Figure 11. Average allele frequency dynamics of simulations where no functional resistance alleles were present by the end of year 5. Each panel lists the number of simulations out of 40 which were included in each group. The opacity of the lines and text is indicative of the blood meal mortality rate for a given group of simulations.

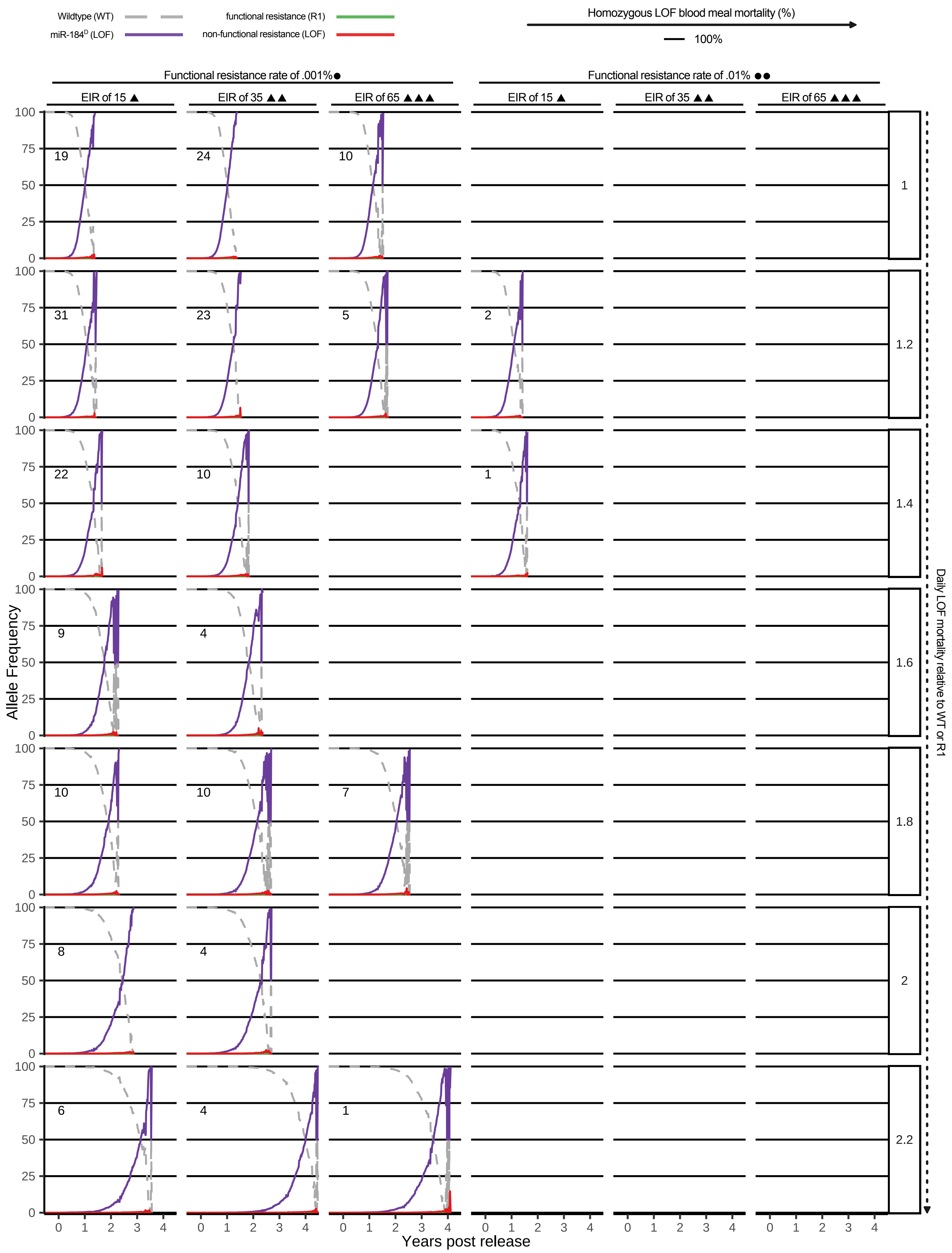

Supplementary Figure 12. Example simulations resulting in the eradication of the local mosquito population. Each panel lists the number of simulations out of 40 where eradication occurred. The opacity of the lines and text indicates the blood meal mortality rate. Single runs are shown instead of the mean due to allele frequency fluctuations near the time of a population crash. No simulation resulted in a population crash when functional resistance was high (0.1%).

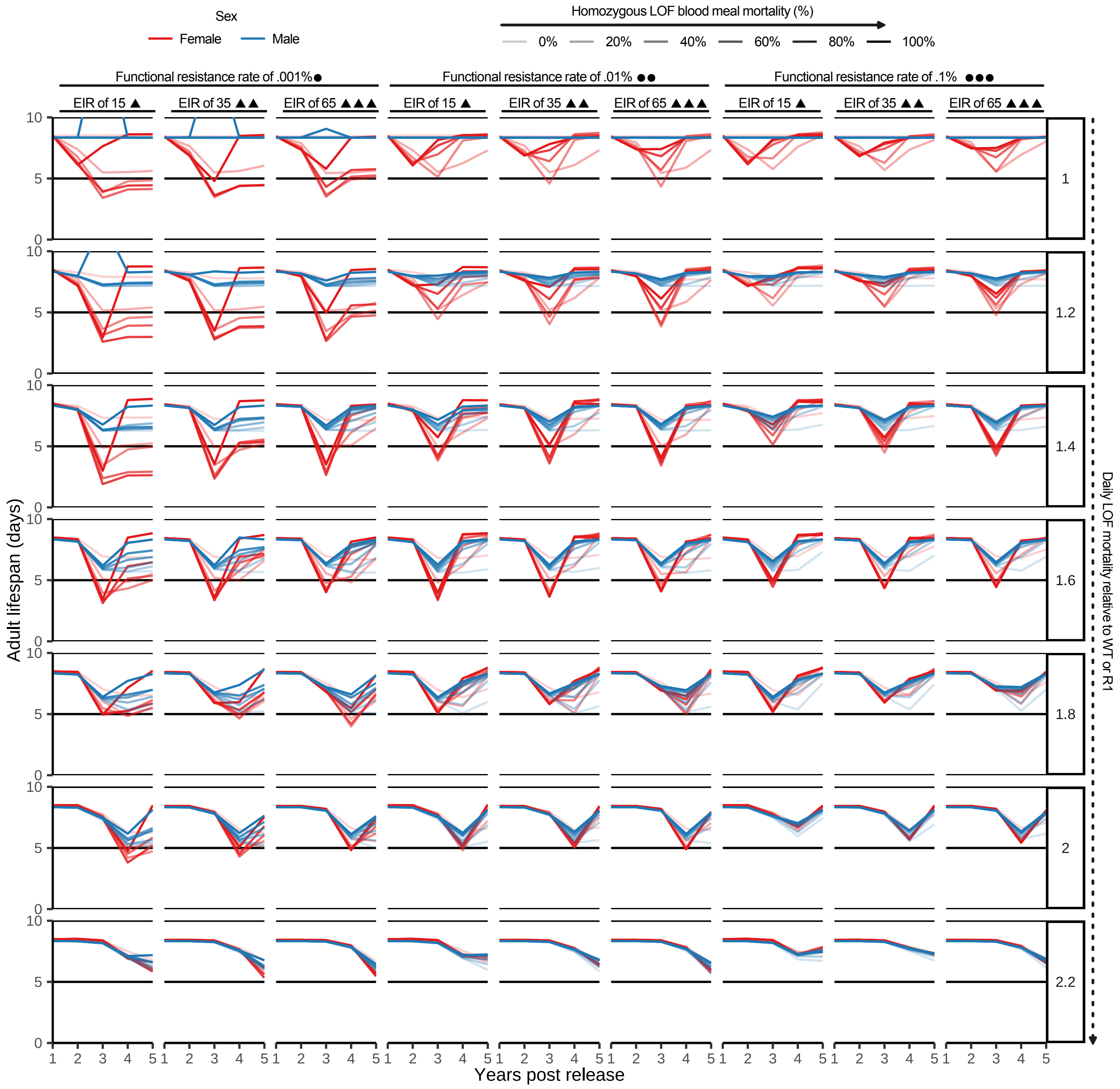

Supplementary Figure 13. Average lifespan of female and male mosquitoes following the release of the drive. Blood meal lethality specifically affects females, whereas daily mortality affects both sexes. The opacity of the lines indicates the blood meal mortality rate. Lifespan is averaged over a year due to seasonal fluctuations. Increased lifespan can occur when density dependence or temporal dynamics of drive suppression result in the overrepresentation of longer-lived individuals.
