# Supplementary material for: A suppression-modification gene drive for malaria control targeting the ultra-conserved RNA gene mir-184
Source: Nat Commun. 2025 Apr 25;16:3923. doi: 10.1038/s41467-025-58954-5 (PMC12032250; doi:10.1038/s41467-025-58954-5)
Supplement: Supplementary file 2 — Description of Additional Supplementary Files [file 41467_2025_58954_MOESM2_ESM.pdf]

Description of Additional Supplementary Files

File Name: Supplementary Data 1
Description: Differentially expressed genes and gene ontology analysis data.
